# Phylogenomics as an effective approach to untangle cross-species hybridization event: A case study in the family Nymphaeaceae

**DOI:** 10.3389/fgene.2022.1031705

**Published:** 2022-11-03

**Authors:** Lin Cheng, Qunwei Han, Fei Chen, Mengge Li, Tiago Santana Balbuena, Yiyong Zhao

**Affiliations:** ^1^ Henan International Joint Laboratory of Tea-oil Tree Biology and High-Value Utilization, Xinyang Normal University, Xinyang, Henan, China; ^2^ College of Tropical Crops, Hainan University, Haikou, China; ^3^ Department of Agricultural, Livestock and Environmental Biotechnology, UNESP, São Paulo, Brazil; ^4^ State Key Laboratory of Genetic Engineering and Collaborative Innovation Center of Genetics and Development, School of Life Sciences, Fudan University, Shanghai, China; ^5^ College of Agriculture, Guizhou University, Guiyang, China

**Keywords:** water lily, Nymphaeaceae, phylogenomics, phylogeny, multi-labeled gene family tree, hybridization, allopolyploid

## Abstract

Hybridization is common and considered as an important evolutionary force to increase intraspecific genetic diversity. Detecting hybridization events is crucial for understanding the evolutionary history of species and further improving molecular breeding. The studies on identifying hybridization events through the phylogenomic approach are still limited. We proposed the conception and method of identifying allopolyploidy events by phylogenomics. The reconciliation and summary of nuclear multi-labeled gene family trees were adopted to untangle hybridization events from next-generation data in our novel phylogenomic approach. Given horticulturalists’ relatively clear cultivated crossbreeding history, the water lily family is a suitable case for examining recent allopolyploidy events. Here, we reconstructed and confirmed the well-resolved nuclear phylogeny for the Nymphaeales family in the context of geological time as a framework for identifying hybridization signals. We successfully identified two possible allopolyploidy events with the parental lineages for the hybrids in the family Nymphaeaceae based on summarization from multi-labeled gene family trees of Nymphaeales. The lineages where species *Nymphaea colorata* and *Nymphaea caerulea* are located may be the progenitors of horticultural cultivated species *Nymphaea* ‘midnight’ and *Nymphaea* ‘Woods blue goddess’. The proposed hybridization hypothesis is also supported by horticultural breeding records. Our methodology can be widely applied to identify hybridization events and theoretically facilitate the genome breeding design of hybrid plants.

## Introduction

Gene flow between population and species is common (Ellstrand and Rieseberg, 2016), including horizontal gene transfer, introgression and hybridization, *etc.* The transfer of genetic material from one population to another can greatly enhance the population’s fitness and adaptation (Lenormand, 2002). Hybridization or allopolyploidy is a large-scale gene flow event, and has now been proved as a common and significant process in the evolution of plants, animals, and fungi (Rieseberg, 1997; Giraud et al., 2008; Soltis and Soltis, 2009; Payseur and Rieseberg, 2016).

**FIGURE 1:**
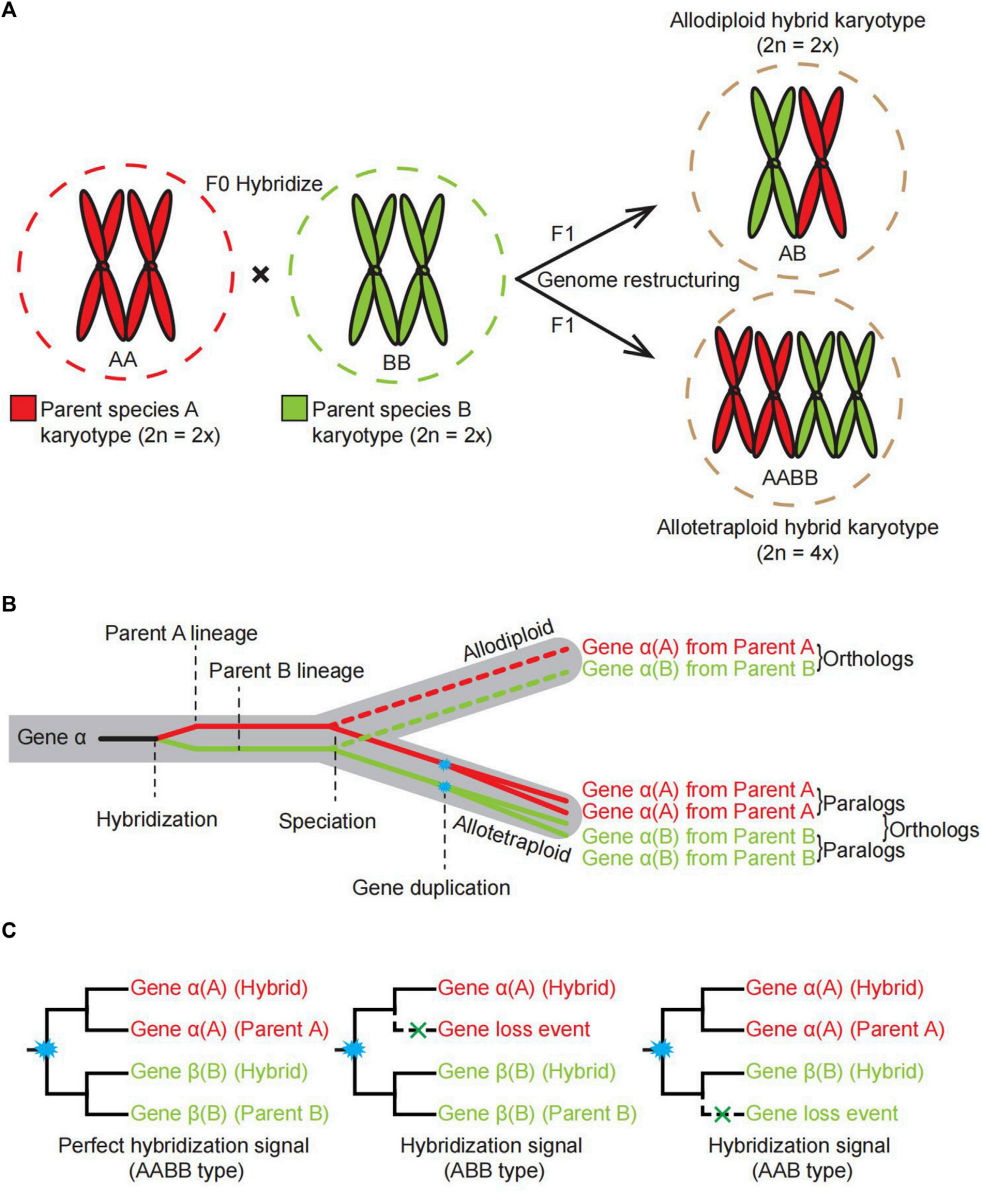
Schematic illustrating the processes of allopolyploidy events, dynamics of karyotype changes and different types of gene family trees considered as hybridization signals. **(A)**. The dynamic karyotype change for offspring of allopolyploids by crossing two diploid parents. **(B)**. Schematic diagram showing the transfer of genes from parental species to offspring through hybridization, as exemplified by gene alpha. **(C)**. A simplified three different gene family tree topologies as signals to support a hybridization event. Cyan asterisks in the node indicate a gene duplication event.

Hybrids inherit genetic information from parental lineages by hybridizing different strains, varieties, or species (Figures 1A,B). There are several types of formation and various characteristics of hybridization events. In terms of ploidy, the progeny of hybridization could be polyploid or homoploid (Figure 1A). Wheat, cotton, and canola are all allopolyploid crops with improved agricultural traits over their diploid counterparts (Rieseberg and Willis, 2007). The sunflower of *Helianthus* is a typical example of homoploid reticulate evolution through hybridization (Paun et al., 2009). In terms of the time scale on which hybridization occurs, there are ancient hybridization evens (paleo-allopolyploidy) and recent hybridization (neo-allopolyploidy); Recently produced hybrid (neopolyploids), often their parental species are extant. In the Brassicaceae family, several rounds of paleopolyploidy have occurred in the most common ancestor of the whole family, but also contain neopolyploids in young evolved lineages, for example, allotetraploid *Brassica napus*. In terms of the number of hybridization events experienced in the process of hybrid species formation, some species have undergone multiple rounds of hybridization events, while others have undergone only one.

The formation of allopolyploidy can be morphed by chromosome doubling in a short time (Rieseberg and Willis, 2007). It has now been confirmed in flowering plants and ferns, and even in animals, allopolyploidy is an important mechanism for new speciation (Paun et al., 2009; Morales-Briones et al., 2018). One explanation for the evolutionary mechanism is that organisms could handle selection pressures with a set of alleles, which allows organisms or allopolyploid populations have more resilient to rigid environments due to the buffering effect of gene duplications (Van-de-Peer et al., 2020) and increase the chances of acquiring new functional genes (Rausch and Morgan, 2005). Compared with their diploid parents, allopolyploids can be adapted to more extreme environmental and climate changes (Fawcett and Peer, 2010; Van-de-Peer et al., 2020; Innes et al., 2021). Homoploidy may also form new species, including one or more chromosomes of a parent or DNA tablet segments integrated into the genome of another parent, leading to the formation of a third species with parental characteristics with a process called introgressive hybridization (Buerkle et al., 2000; Arabaci et al., 2021). Gene rearrangement and redistribution of hybrid progenies may occasionally form a new population that is homozygous to certain chromosomal sterile factors (Rieseberg, 1997). These new hybrid populations would be fertile, stable, and chromosomally identical to the parents, because of the isolation of chromosomal sterility, which made reproductive isolation from the parents (Buerkle et al., 2000; Gross and Rieseberg, 2005). It is also suggested that interspecific hybridization, especially introgression and gene flow, is preferentially selected and leads to adaptive evolution within the populations (Mallet, 2005; Soucy et al., 2015). Therefore, hybridization has been widely used in crop breeding, such as super-hybrid rice. As a result of improving morphological traits and utilizing inter-subspecific heterosis, much progress has been made in developing super hybrid rice varieties (Yuan, 2017). It is of great significance to establish an efficient method for detecting organisms’ hybridization events.

During most eukaryotes’ sexual reproduction, the number of chromosome sets during meiosis alternates between halving and restoring the original ploidy level during fertilization (Mercier et al., 2015). The process of hybridization between two species involves the separation of the homologous chromosomes of the germ cells in both species, followed by returning numerous chromosomes to both parents after fertilization, thereby maintaining genomic stability. A homoploid hybrid species (a diploid in this case) arising through hybridization between parent A and parent B contains one chromosome complement of each parental species (Figure 1A). Although allopolyploid formation involves similar hybridization between A and B, it results in chromosome doubling (Soltis and Soltis, 2009) (Figure 1A). The resultant allopolyploid species contains the genes with different copies from both parental species (Figure 1B, illustrated by an example gene *alpha*). Therefore, the multiple labeled gene family trees could be the signal to support hybridization events (Figure 1C).

When hybridization occurs in ancient times or multiple parental lines are involved in hybridization history, it is generally more difficult to detect hybridization signals. For instance, the cultivated peanut *Arachis hypogaea* have identifiable subgenomes that could easily trace from two parental diploid ancestors, *Arachis duranensis* and *Arachis ipaensis* (Bertioli et al., 2016). The cultivated wheat is derived from three ancestral diploid species (Dubcovsky and Dvorak, 2007; El Baidouri et al., 2017) and genome sequencing revealed that modern cultivated octoploid strawberry *Fragaria ananassa* probably has four diploid parents (Edger et al., 2019). Walnut *Juglans regia* has also shown weak signals to be ancient hybrids (Zhang et al., 2019).

Identifying hybridization events is important for our understanding of speciation, adaptation and heterosis, so several methods have been proposed to identify allopolyploidy events. The GRAMPA method is a relatively new approach to determining whether an allopolyploidy event has occurred (Gregg et al., 2017). However, the results of GRAMPA analyses can be easily affected by the number of represented species (Koenen et al., 2021; Zhao et al., 2021) and can yield ineffective statistical information. PhyloNet was proposed for analyzing and reconstructing reticulate evolutionary relationships (Than et al., 2008), but it has the disadvantage that its processing is slow for large-scale data. Phylogenomics is a powerful tool for tracing the parental lineages of a hybrid since homoploids and allopolyploids contain genetic information from both parents. Compared with other published methods, the phylogenomics approach identifies hybridization more directly by comparing multi-labeled gene trees with species trees, obtaining gene family trees that support the hybridization hypothesis, and finally summarizing the hybridization signal (Figure 1C).

Water lily is a common name for all species categorized into the order Nymphaeales (Saarela et al., 2007; Chen et al., 2017), divided into three families: Hydatellaceae, Cabombaceae, and Nymphaeaceae (Group, 2016). Many reputed artists worldwide, including the French impressionist Claude Monetits, have been captivated by the aesthetic beauty of these flowers and their colors (Zhang et al., 2020). The orders Amborellales, Nymphaeales, and Austrobaileyales, collectively termed the ANA grade, diverged as separate lineages from a remaining angiosperm clade (Group, 2016). Nymphaeales comprise eight genera (*Trithuria*, *Cabomba*, *Brasenia*, *Barclaya, Euryale*, *Nuphar, Victoria*, and *Nymphaea*), containing 74 species (angiosperm phylogeny website, version 14, http://www.mobot.org/MOBOT/Research/APweb/welcome.html). Nymphaeales contain the highest number of species among the three aforementioned early-diverging angiosperm orders, including economically important species. Due to their debated relationship with *Amborellales*, the phylogeny of the Nymphaeales has been extensively studied by numerous recent studies focusing on the phylogenetic relationships among the five subgenera as well as *Victoria* and *Euryale*, which are still largely unclear (Borsch et al., 2008; Biswal et al., 2012).

In addition to their ornamental value in horticulture, water lilies are an important model plant because of their short life cycles and large seed numbers (Chen et al., 2017). *Nymphaea* hybrids and allopolyploids have been formed recently through artificial hybridization breeding programs, according to the international waterlily and water gardening society (https://www.internationalwaterlilycollection.com/). In particular, the genome of *Nymphaea colorata* has been released (Zhang et al., 2020) and *N. colorata* has a relatively small genome size (2n = 28 and approximately 400 Mb) and blue petals that make it popular in breeding programs. The beautiful blue petals of *Nymphaea colorata* represent an economically important trait such that its gene(s) have been introduced into other cultivars. For example, *N. colorata* is one of the parents for the following cultivars: *N.* ‘Woods Blue Goddess’, *N.* ‘Midnight’ (www.internationalwaterlilycollection.com). In particular, *Nymphaea* ‘Midnight’ is a George H. Pring waterlily. It is a double deep purple star with slightly flecked pads. It was cultivated in 1940 and one of its parents is *Nymphaea colorata*. It was one of the first known hybrids to have the stamen become small petals. *Nymphaea* ‘Woods Blue Goddess’ is a tropical waterlily created by John Wood. Its date of origin is 1989 and has sky blue star-shaped petals and green pads. It is one of parentage is *Nymphaea colorata*. Generally, *Nymphaea colorata* is a common parental species for multiple *Nymphaea* hybrids. Therefore, *Nymphaea colorata*, with beautiful pure blue petals, is a valuable germplasm resource for horticulture.

The progress of research of phylogenomics and phylotranscriptomics has been greatly accelerated by the rapid development of next-generation sequencing. Analyses using nuclear genes successfully resolved relationships among major angiosperm lineages (Guo et al., 2020; Zhang et al., 2020; Cheng et al., 2022; Huang et al., 2022; Zhang et al., 2022). Mutations of sequences are considered to be directionless; not every gene evolves in a direction that is the same as the evolutionary direction of the speciation. Many previous studies have favored using more conserved low-copy nuclear genes that have proven effective in resolving reticulate phylogenetic relationships (Zhang et al., 2012; Zeng et al., 2014; Cai et al., 2019; Guo et al., 2020; Zhang et al., 2020; Cheng et al., 2022; Huang et al., 2022; Zhang et al., 2022). Current methods for studying hybridization mainly use genome-wide large-scale genes, which inevitably introduces a lot of noise. In this study, the hybridization events in two hybrid water lilies have been characterized from multi-labeled gene family trees by using conserved low-copy nuclear genes. Using genome and transcriptome sequencing along with comparative phylogenomic analyses, the genetic contents of two hybrids were successfully characterized and mapped to parental lineages.

## Results

### Phylogenomics approach confirmed a robust nymphaeales phylogeny by integrating multi-labeled gene trees

**FIGURE 2:**
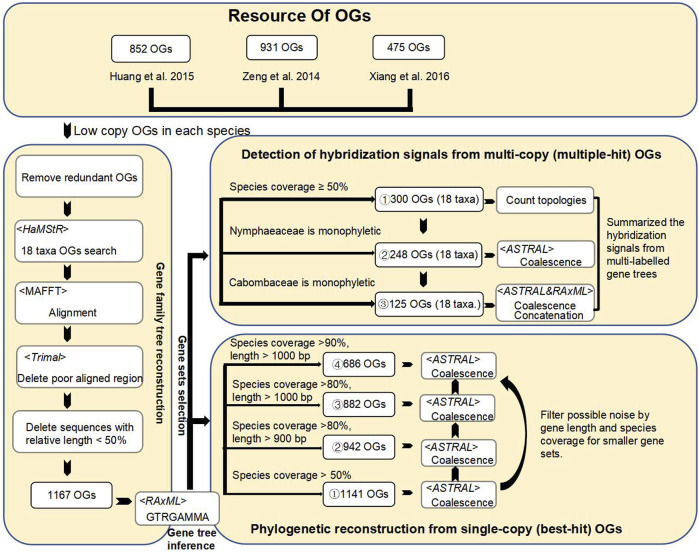
The workflow for orthogroups identification, filtering to improve phylogenetic signals and procedures to identify signals from multi-copy of orthologous gene sets for hybridization event detection. A total of filtered 1,141 low-copy nuclear genes from the previous study (Zhang et al., 2020) were used to reconstruct the Nymphaeales phylogenetic framework. A total of four selected low-copy gene sets (1,141, 942, 882, and 686 OGs) by gene length and species coverage for coalescence tree analyses. The multi-copy gene set was obtained by searching for all homologs of the species corresponding to these low-copy nuclear genes. To detect hybridization signals, two multi-labeled gene sets (248 and 125) were selected based on the presence of genes from outgroup *Amborella*), monophyly in the family (Nymphaeaceae and Cabombaceae) and species coverage in major phylogenetic groups. Finally, the hybridization signals were summarized into a species tree by the coalescent method from multi-labeled gene family trees with 17 sampled water lilies and one outgroup.

Accurate and stable phylogeny is the basis for tracing the hybridization history. The occurrence of polyploidy events in the ancestor of the water lilies has greatly increased the difficulty of identifying putative orthologs for phylogenetic analyses. Our study reanalyzed the Nymphaeales phylogeny with 17 water lilies and 1,141 low-copy nuclear genes (Figure 2) from a previous study (Zhang et al., 2020). Three *Nymphaea* hybrids (*Nymphaea* ‘Paramee’, *Nymphaea* ‘Choolarp’ and *Nymphaea* ‘Thong Garnjana’) with messy hybridization histories were not included in phylogenetic and hybridization analyses. A total of 1,141 conserved nuclear genes were used to identify both single-copy (best-hit) and multi-copy (multiple-hits) for further phylogenetic analyses.

As an early diverging lineage from all other extant flowering plants, the Nymphaeales order contains three families: Nymphaeaceae, Cabombaceae, and Hydatellaceae (Group, 2016). However, the monophyly of Nymphaeaceae and the status of the other two families remain controversial (Biswal et al., 2012; Gruenstaeudl et al., 2017). Our results corroborate that Nymphaeaceae is monophyletic, represented by four genera, confirmed by plastid genomes, and Cabombaceae containing two genera of *Cabomba* and *Brasenia* is determined to be a sister to Nymphaeaceae (He et al., 2018). We further reconstructed a robust Nymphaeales phylogeny which is consistent with the previous study (Zhang et al., 2020). Compared to the previous phylogenomic study (Zhang et al., 2020), this study using only Nymphaeales representatives and low-copy and multi-copy gene markers with several gene sets further confirmed the phylogenetic framework of Nymphaeales.

**FIGURE 3:**
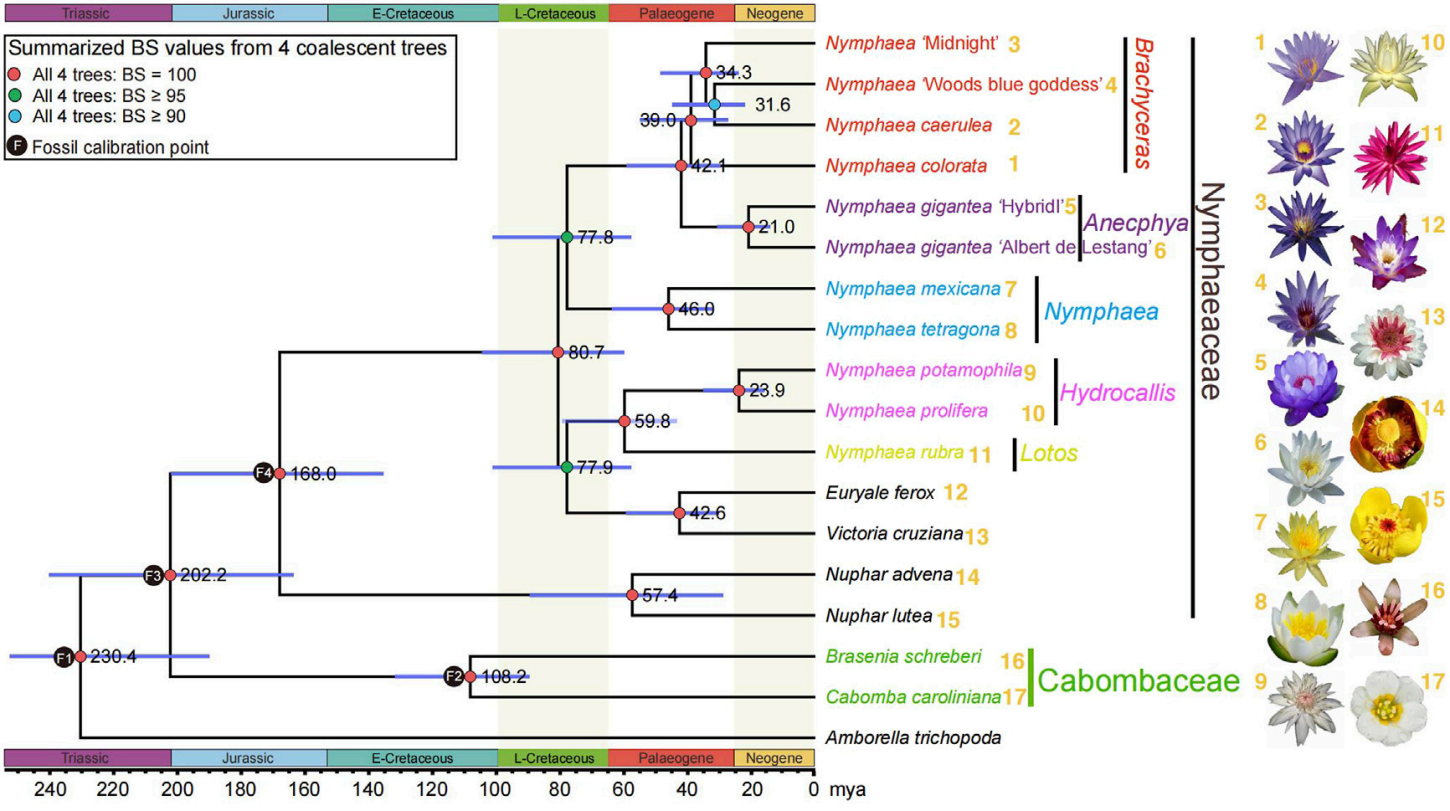
A summarized Nymphaeaceae phylogeny from four coalescent trees with corresponding bootstrap values mentioned on the left of nodes and divergence times inferred by treePL. Red circles indicate maximum (100%) bootstrap support in all four analyses. Green circles indicate at least 95% bootstrap support in all four coalescence analyses from 1,141, 942, 882, and 686 gene sets. Cyan circles indicate at least 90% bootstrap support in all four analyses. The right side of the species name shows the flowers of the 20 sampled water lilies from our previous study (Zhang et al., 2020).

As shown in Figure 2, four step-wise filtered data sets of low copy-number orthogroups (OGs) (1,141 OGs, 942 OGs, 882 OGs, 686 OGs) were used to reconstruct phylogenetic relationships using the coalescent method based on species coverage and length of aligned matrixes (see Methods and Figures 2, 3). Among the four sampled Nymphaeaceae genera, *Nuphar* was determined to be the earliest diverging lineage, in agreement with previous phylogenetic results of chloroplastid genomes with 66 plastid protein codon genes from 13 Nymphaeaceae species (Gruenstaeudl et al., 2017; He et al., 2018). However, the *Nympheae* genus was not determined to exhibit monophyly, with the nested sister pairs of *Victoria* and *Euryale*, forming a sister group to that of the *Hydrocallis* and *Lotos* subgenera, which first diverged in *Nymphaea* (López-Caamal and Tovar-Sánchez, 2014). The limited sampling in previous plastid phylogenetic studies resulted in *Victoria* and *Euryale* being nested into the genus *Nymphaea* (Gruenstaeudl et al., 2017; He et al., 2018). The combination of *Victoria* and *Euryale* formed a sister group with *Nympheae jamesoniana*. (Gruenstaeudl et al., 2017; He et al., 2018). *Nymphaea* species are widely distributed into five subgenera, with three subgenera, including *Nymphaea*, *Anecphya*, and *Brachyceras*, diverging successively.

**FIGURE 4:**
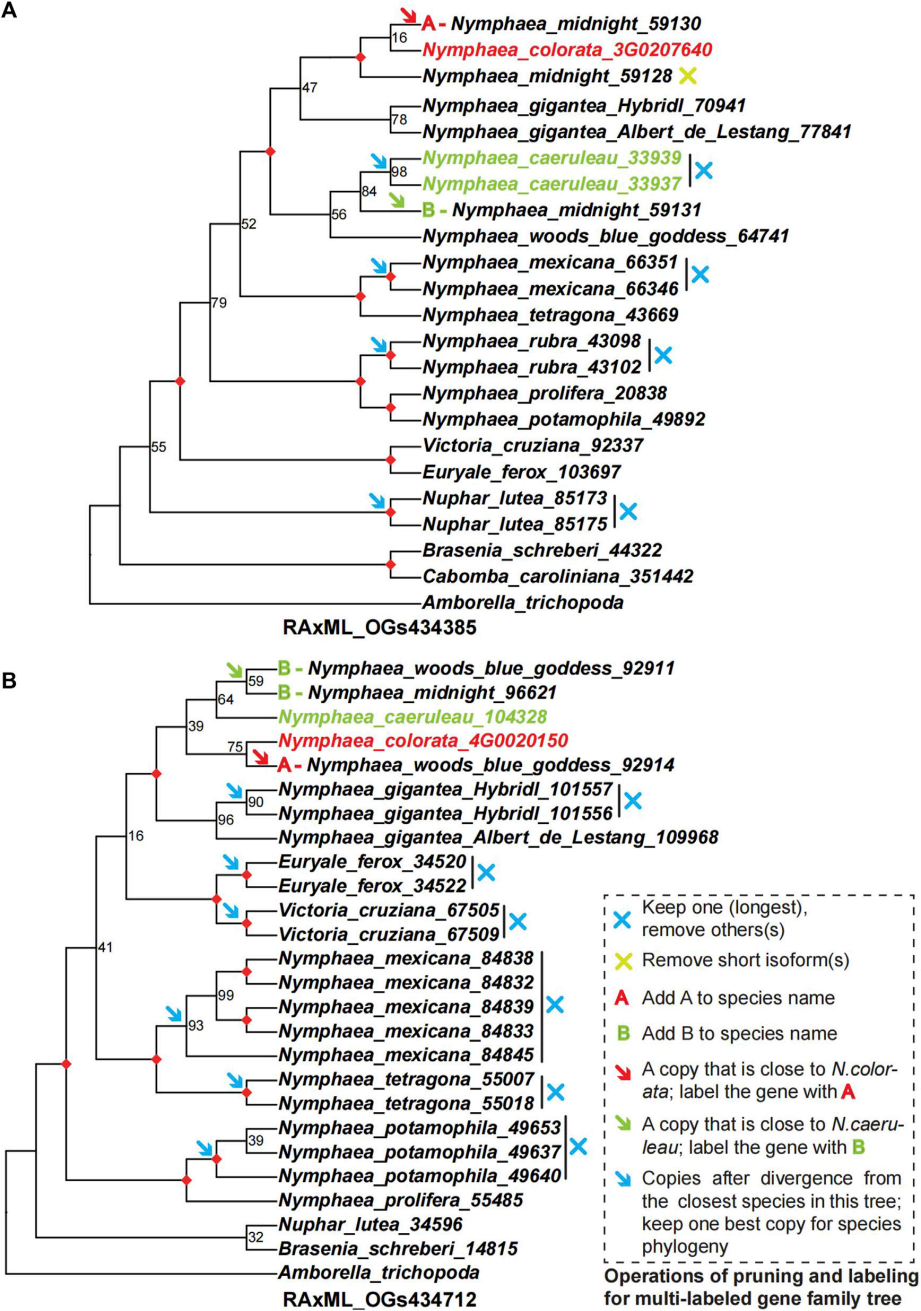
The examples of two gene family trees support a hybridization event and processing for pruning and labelling the multi-copy gene trees to multi-labeled gene trees as hybridization signals. **(A,B)** show two multi-copy gene family trees numbered RAxML_OGs434385 and RAxML_OGs434712 to illustrate operations of pruning and labelling of multi-labeled gene family trees. These two gene family trees can be used to support the hybrids of *Nymphaea* ’Midnight’ and *Nymphaea* ’Woods blue goddess’ from parental lineages of *Nymphaea colorata* and *Nymphaea caerulea* by hybridization. The multi-copy ML phylogenetic trees were inferred from nucleotide sequences with the nucleotide substitution model GTRGAMMA by RAxML. The number at each node is the bootstrap support value and the maximal (100%) support value is marked by a red diamond. The redundant paralogs or isoforms were removed from the multi-copy gene family trees based on the principle described inside the dashed box on the right of Figure **(B)**. The modified gene family trees were then transferred to multi-labeled gene family trees, labelling the A/B for two paralogs of hybrids derived from two parental lineages. These multi-labeled gene family trees maintain a most plausible single copy for all species, except for the hybrids, which contain multiple copies with labels. In addition, multi-labeled gene family trees were used for coalescence and supermatrix ML analyses to detect hybridization events.

**FIGURE 5:**
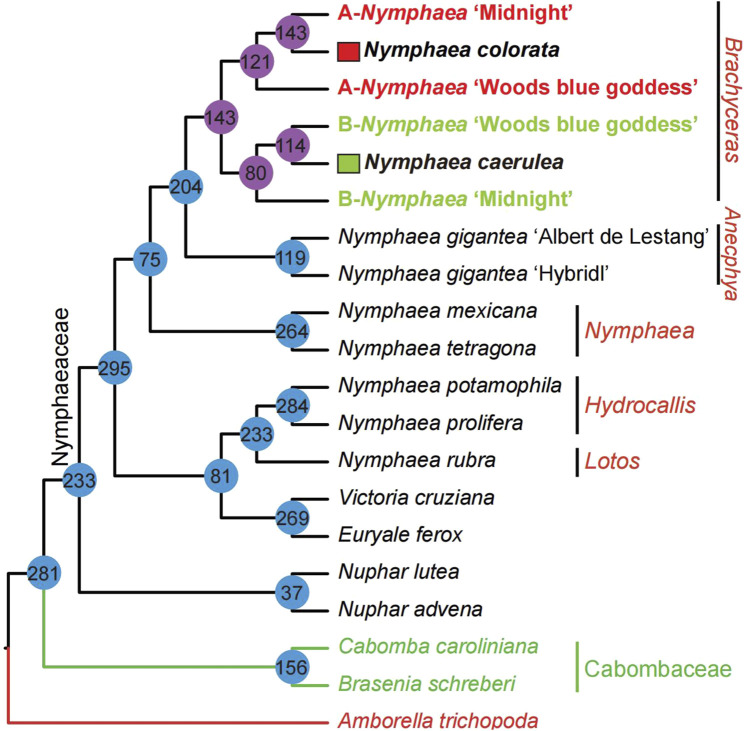
The topology statistics of multi-labeled gene family trees mapping to the Nymphaeales phylogeny. A total of 300 multi-labeled gene family trees were used to count the topology and the detailed gene selection and processing pipeline were described in Figure 2. The number inside the circle on the phylogenetic tree nodes shows the number of gene families that support the phylogenetic relationship.

**FIGURE 6:**
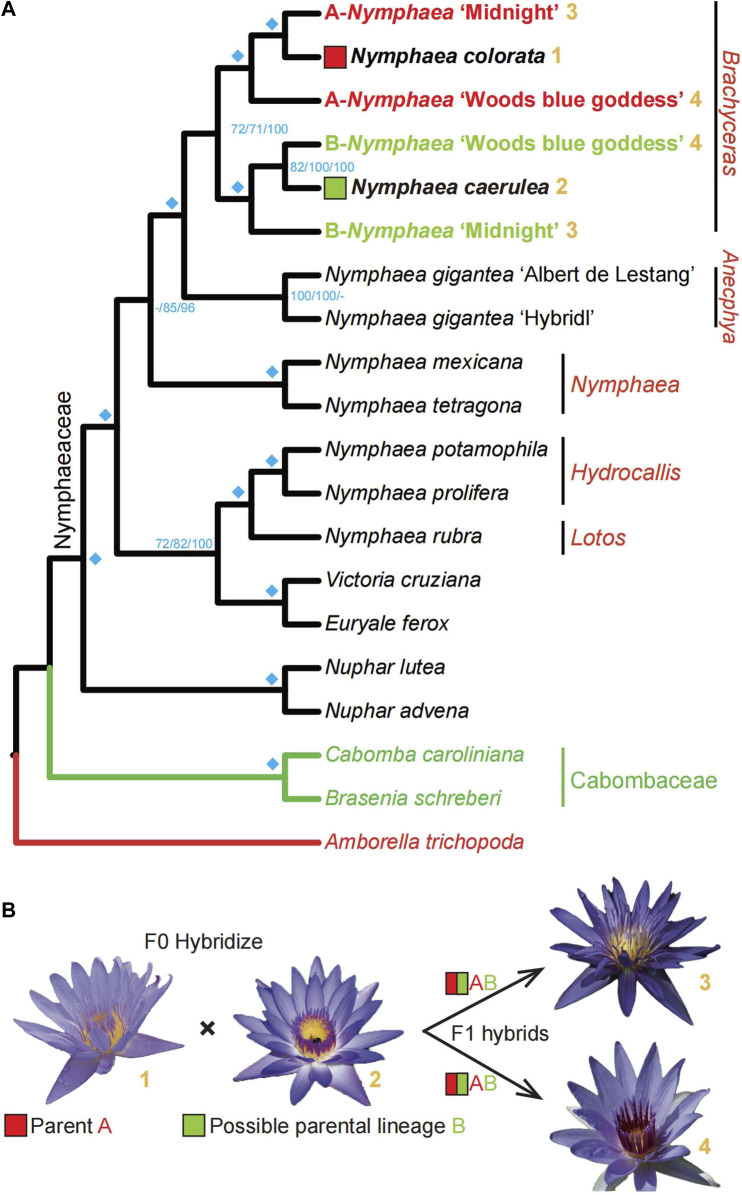
High-quality *Nymphaea colorata* genome and other transcriptomes facilitate a survey of the hybridization histories of *Nymphaea* ’Midnight’ and *Nymphaea* ’Woods blue goddess’. **(A)**. The summarized phylogeny from three multi-labeled phylogenetic trees with corresponding bootstrap values on the nodes. In that order, the three supports value on this summarized tree nodes come from two coalescent trees of 125 and 248 genes and one supermatrix maximum-likelihood tree of 125 genes. The cyan diamonds on the left of nodes indicate that all three bootstrap value are maximal (100). *Nymphaea colorata* and the lineage of *Nymphaea caerulea*, are regarded as the likely hybridization progenitors of the cultivated garden plant, *Nymphaea* ’midnight’ and *Nymphaea* ’Woods blue goddess’, based on summarization from multi-labeled gene family trees of Nymphaeales. Bold yellow numbers coded the four water lilies involved in hybridization events after the species names; the flowers of the four sampled water lilies used in B proposed the hybridization hypothesis. **(B)**. The diagram shows the possible hybridization events identified by the phylogenomic method. The lineages where species *Nymphaea colorata* and *Nymphaea caerulea* are located may be the progenitors of horticultural cultivated species *Nymphaea* ’midnight’ and *Nymphaea* ’Woods blue goddess’.

To further exclude the effect of paralogs, we used a total of 300 multi-copy gene family trees to reconstruct Nymphaeales phylogeny (see Methods and Figures 2, 4 for the procedure of pruning and labeling multi-labeled gene family trees). We counted the topologies of these 300 multi-labeled gene families (Figure 5) and did further coalescent and supermatrix ML analyses of two gene sets (Figures 2, 6). In summary, the phylogenetic analyses of these carefully manually checked multi-labeled gene family trees also yielded consistent phylogenetic relationships with the phylogeny inferred from 1,141 low-copy nuclear genes. The multi-labeled gene family trees also support that *Nympheae* is not a monophyletic genus; We also detected 81 gene family trees supporting the embedding of *Victoria* + *Euryale* into the genus *Nympheae* from the topology statistics of multi-labeled gene family trees (Figure 5). Our results suggested *Victoria* and *Euryale* require a new taxonomic revision. The confirmed robust Nymphaeales phylogeny was further used as the phylogenetic framework for reconciliation and mapping for identifying hybridization events.

Morphological studies are of great value to understanding plant phylogeny and evolution. Unfortunately, the convergent evolution of different species trying to survive in the same environment (aquatic lifestyle in water lilies) will lead to different degrees of similarity in their morphological traits, which undoubtedly increases the difficulty of morphological classification significantly. In the last decade, several systematic studies of the water lily family were conducted by combining morphological and molecular evidence. Based on molecular and morphological evidence, multiple studies support *Brachyceras*, *Anecphya*, *Nymphaea*, *Hydrocallis*, *Lotos* as a monophyletic subgenera, respectively, in agreement with our results (Borsch et al., 2007; LÖHNE et al., 2007; Borsch et al., 2008; Taylor, 2008; He et al., 2018). The combined morphological and molecular analyses conducted by Borsch et al. (2008) suggest that the genus *Nymphaea* is paraphyletic, with the subgenus *Nymphaea* being sister to a clade comprising the other subgenus and the *Euryale*-*Victoria* clade. Additionally, Distribution data and fossil records were used to reconstruct ancestral ranges of Nymphaeales, supporting the common ancestor of *Hydrocallis*, *Lotos*, *Victoria* and *Euryale* originated in Eurasia. Nuclear phylogenetic studies support *Nymphaea* as paraphyletic genera with embedding *Euryale* and *Victoria*, which is consistent with previous studies (Borsch et al., 2007; LÖHNE et al., 2007; Borsch et al., 2008; He et al., 2018). The observations of morphologies (e.g., ovule) suggest that the *Nymphaea* diversified extensively and the characters studied clearly show no evidence to support the hypothesis of a monophyletic genus (Borsch et al., 2007; Zini et al., 2015). The vegetative and leaf architectural characters and 62 morphological support *Anecphya* + *Brachyceras* and *Hydrocallis* + *Lotus* is the sister group to each other (Borsch et al., 2008; Taylor, 2008), which is consistent with our results.

### Origination of genera from late cretaceous in Nymphaeaceae

Molecular clock-based estimation of divergence times for many lineages provided possible geological and environmental contexts for Nymphaeaceae evolutionary studies. The crown group of the waterlily family is dated 168.0 million years ago (mya) in the Jurassic era, which is in alignment with the confidence interval of the estimated time in a previous study (Zhang et al., 2020). After a long evolutionary time range from the origination of the waterlily family, the *Nymphaea* crown group with diverse species first originated during the late Cretaceous era (80.7 mya), and the most recent ancestors of the earliest divergent, *Nuphar*, has been estimated to be 57.4 mya. The *Euryale* and *Victoria* genera diverged in the Palaeogene era (42.6 mya) (Figure 3). The estimated time of *Nuphar* crown groups was found to be different from that in the previous study; however, the time confidence interval (CI) for *Nuphar* with a wide range was still found to overlap with the previously estimated time CI (Zhang et al., 2020), suggesting that the time accuracy of *Nuphar* could probably be reevaluated by increasing sampling.

### Tracing parental lineages of waterlily hybrids based on phylogenomics method

*Nymphaea* ‘Midnight’ and *Nymphaea* ‘Woods blue goddess’ are the two hybrids that retained two duplicates in 300 gene trees with nine groups, each including at least one taxa to trace back their phylogenetic positions. In our sampling, one copy of *Nymphaea* ‘Midnight’ was a sister to *Nymphaea colorata* and the other copy grouped with the sister pairs of *Nymphaea caerulea* and *Nymphaea* ‘Woods blue goddess’. The two copies of *Nymphaea* ‘Woods blue goddess’ were sisters to *Nymphaea caerulea* and clustered with a pair of *Nymphaea colorata* and *Nymphaea* ‘Midnight’. As illustrated in Figure 4, two copies of each hybrid derived from different progenitors were partitioned and grouped along with two distinct lineages. In order to detect hybridization events by simplifying gene trees and reducing them single representative tips, the rule of monophyly was first applied to each species with multiple copies to identify the longest tips as representatives (as indicated by red arrows in Figure 4). In order to focus on the two hybrids, *Nymphaea* ‘Woods blue goddess’ and *Nymphaea* ‘Midnight’, their representative tips were designated with the prefix “A” and “B” that grouped with *Nymphaea colorata* and *Nymphaea caerulea*, respectively, except the removal of paralogs (as indicated by deep blue crosses in Figure 4).

To better understand the topologies of 300 multi-labeled gene family trees mentioned above, we compare them with the species tree to obtain the number of gene families supported for each node (Figure 5). The number of multi-labeled gene family trees supporting the parentage of *Nymphaea colorata* clustered together with two hybrids A-*Nymphaea* ‘Midnight’ and B-*Nymphaea* ‘Woods blue goddess’ is 143 and 121, respectively. We also observed 114 and 80 multi-labeled gene trees supporting B-*Nymphaea* ‘Woods blue goddess’ and B-*Nymphaea* ‘Midnight’ clustered with another possible parental lineage *Nymphaea caerulea*, respectively (Figure 5). Furthermore, we used coalescence and supermatrix ML methods to summarize these hybridization signals from multi-labeled gene family trees (Figure 6A). The coalescence and ML trees strongly support the hybrid origins of *Nymphaea* ‘Midnight’ and *Nymphaea* ‘Woods blue goddess’, with their possible parentage of *Nymphaea colorata* and *Nymphaea caerulea* lineage based on limited sampling size (Figure 6). Confirming from the clear cultivation history of cultivated hybrid origins by horticulturalists (https://iwgs.org/), *Nymphaea colorata* is a common progenitor that hybridizes with *Nymphaea capensis* var. *Zanzibariensis* and *Nymphaea ampla* to yield *Nymphaea* ‘Midnight’ and *Nymphaea* ‘Woods blue goddess’, respectively (https://www.internationalwaterlilycollection.com/) (Zhang et al., 2020).

Our study did not include *Nymphaea capensis* and *Nymphaea ampla* for our analyses. It is not confirmed that they are the extant parents of *Nymphaea* ‘Midnight’ and *Nymphaea* ‘Woods blue goddess’ through the breeding program, respectively. Currently, no published studies have resolved the relationship between these water lilies including *Nymphaea capensis*, *Nymphaea ampla* and *Nymphaea caerulea*. We speculated that the missing parents *Nymphaea capensis* var. *Zanzibariensis* and *Nymphaea ampla* are more closely related to *Nymphaea caerulea*, so we could detect another gene copy of the two hybrids clustered with the lineage where *Nymphaea caerulea* is located. Generally, species of parentage can be detected more precisely with more samples included through hybridization. Further research in Nymphaeaceae requires the integration of more representative water lilies to identify allopolyploidy events.

## Discussion

In the early stages of angiosperm phylogenetic studies, organelle (chloroplast, mitochondrial) genes or their gene spacer regions were widely used because they were easily accessible. The chloroplast is a unique organelle in plants, and its cyclic genome DNA is divided into large single copy (LSC) and small single copy (SSC) regions by two inverted repeat sequences (IR). Although the copy number of chloroplast DNA (cpDNA) varies among species, the gene composition and arrangement are similar, and the number of genes is almost the same. Meanwhile, chloroplast gene sequences are relatively conservative and can be easily amplified and cloned, so they are widely used in angiosperm phylogenetic studies (Olmstead and Palmer, 1994). Some conserved nuclear genes are easy to clone and align, and more phylogenetically informative than widely used organellar genes (Zhang et al., 2012). The phylogenetic trees of angiosperms constructed based on different chloroplast single genes often diverge from each other, and the support rate of many branches is not high, which is mainly due to the short sequences of single genes and too few informative loci, resulting in stochastic errors (Rokas et al., 2003; Delsuc et al., 2005; Jeffroy et al., 2006). Multiple chloroplast genes, intergenic regions or even entire chloroplast genomes could not resolve reticulate phylogenetic relationships in previous angiosperms phylogenetic studies (Group, 2016; Li et al., 2021). The development of sequencing technology has made nuclear genome and transcriptome sequencing efficient and rapid. The cost of sequencing has been dramatically reduced, which provides a solid technical basis for obtaining nuclear gene data of multiple species and will make it an important trend to combine a large number of genes and even whole genome data to study the phylogenetic relationships of angiosperms. Identification of orthologous genes is an essential prerequisite for constructing phylogenetic relationships based on nuclear genes. Not only did paleopolyploidy event occurred in the ancestors of the Nymphaeales, but the entire angiosperms have likely undergone multiple rounds of paleopolyploidy events (Ren et al., 2018; Zhang et al., 2020). Although whole-genome doubling generates a large number of duplicated genes (paralogous), the occurrence of paleopolyploidy events is subsequently accompanied by genomic rearrangements and substantial gene loss, and we are still able to identify sufficient effective single- or low-copy nuclear genes for phylogenomic studies (Guo et al., 2020; Zhang et al., 2020; Zhao et al., 2021; Cheng et al., 2022; Huang et al., 2022; Zhang et al., 2022). In our study, in order to minimize the effects of the hidden paralogues and identify putative orthologues, the aforementioned OGs were carefully filtered based on species coverage and gene lengths to identify 1,141 OGs and 942 OGs, respectively. For filtering conditions with a higher species coverage ratio and gene length, two orthologue groups of 882 OGs and 686 OGs were further obtained. In addition, in order to obtain a more accurate and highly supported species relationship, both low-copy and multi-copy of orthologous genes were used for phylogenomic analyses, and yielded a consistent phylogenetic topology. We used 1,167 low-copy nuclear genes and successfully resolved the phylogenetic relationships of Nymphaeales. To exclude the interference of paralogous genes, we identified multiple homologous genes for these low-copy nuclear genes from each species, and obtained the true orthologs by manually deleting the paralogs from the multi-copy gene family trees. The Nymphaeales phylogeny reconstructed from orthologs by excluding paralogs (Figure 6) is consistent with the phylogeny yielded from 1,167 low-copy nuclear genes (Figure 3).

At the family and order level, Nymphaeaceae phylogenies have mostly been supported by analyses using plastid genome sequences (Qi et al., 2018), including studies with extensive taxon sampling that represented most families, albeit with 77 plastid sequences (Gruenstaeudl et al., 2017). The monophyly of Nymphaeaceae has also been inferred by using fast evolving and non-coding chloroplast markers in 70 species, which suggested several alternatives for the placement of *Nuphar* (Löhne et al., 2010; Christenhusz et al., 2016). Another study failed to convincingly support the monophyly of the Nymphaeaceae family by using a combined approach of gene tree and species tree based on *matK* and ITS2 (Biswal et al., 2012). In addition, five complete chloroplast genomes and 66 protein-coding genes were used to infer relationships of Nymphaeaceae (He et al., 2018). However, conflicts or poorly established relationships remain within the family. Here, we reconstructed a robust nuclear phylogeny for the Nymphaeaceae family. Our results strongly supported the monophyly of Nymphaeaceae. However, *Victoria* and *Euryale* are interspersed among *Nymphaea* species, suggesting the paraphyletic group of *Nymphaea* requires further taxonomic revision.

Nuclear protein-coding genes are important for many diverse functions, representing the tremendous majority of the cellular genome and providing markers to track evolution. However, only a few nuclear genes have been used to resolve the relationships of Nymphaeaceae thus far. On the other hand, different nuclear genes could lead to different topologies for phylogeny analysis (Huang et al., 2016a). For instance, short or fragmentary gene sequences can result in incorrect gene tree estimations because of lacking phylogenetic signals or large amounts of missing data (Qi et al., 2018). Moreover, gene duplication can lead to the inclusion of paralogs to the wrong gene tree topologies (Huang et al., 2016b). Therefore, it is important to exclude such misguided sequences from the phylogenetic analysis matrices by using orthologous nuclear genes to avoid noise (Zhang et al., 2012). The phylogeny presented here is robust for relationships among four genera of Nymphaeaceae, having undergone multiple tests and analyses using coalescent and supermatrix ML methods. These results include well-established relationships not only among the families, but also for the four Nymphaeaceae genera, illustrating the effectiveness of using conserved low copy-number nuclear genes for phylogenetic reconstruction. Our results align well with relatively well-supported previous relationships determined using chloroplast genomes (He et al., 2018). Our topology of the major clade is also consistent with another recently reported phylogeny of the water lily genome using transcriptome data sets (Zhang et al., 2020).

In this study, 15 sampled water lilies belonging to Nymphaeaceae, two species of Cabombaceae, along with *Amborella trichopoda* as the outgroup were collected for phylogenetic analyses. We analyzed more than 1,000 low-copy nuclear candidate marker genes and reconstructed a robust and consistent Nymphaeaceae phylogeny strongly supported by multiple phylogenetic analyses. Furthermore, the topology here includes well-supported relationships among the four genera. Most importantly, this study provides the first insight into nuclear phylogenomics in Nymphaeales by integrating both single-copy and multi-copy gene family trees.

Water lilies are among the top two leading aquatic ornamentals alongside sacred lotus (*Nelumbo*, order Proteales). Their aesthetic beauty has captivated many artists, including the French impressionist Claude Monet who painted more than 250 oil paintings of water lilies (http://iwgs.org/invasive-species/, accessed 30 June 2018) (Zhang et al., 2020). Here, we developed a novel method to identify hybridization events based on phylogenomics analyses. This method allows us to identify the parental lineages of allopolyploid species resulting from hybridization by using next-generation sequencing. It also enables us to precisely count the topologies of multi-labeled gene family trees for supporting a hybridization event. However, the reconciliation method has been explored on reticulated phylogenies before (Yu et al., 2013; To and Scornavacca, 2015; Gregg et al., 2017), this is the new method that performs these types of analyses in the context of a robust phylogenetic framework and is applicable to identify large-scale gene flow events, such as introgression or horizontal gene transfer. Identification of hybridization events by this method in Nymphaeaceae ensures that the results inferred from our method align with the breeding records of horticulturalists. The basis for the feasibility of our newly proposed approach is that allopolyploidy produces offspring that carry genetic material from both parents or parental lineages and the paralogs formed a gene duplication event in gene family trees can unveil the hybridization history of the hybrids. However, there are prerequisites for using the phylogenomics approach to detect hybridization events, and in order to identify more accurate parental progenitors, data from both the hybrids and the progenitors need to be included in the analysis.

In the last decade, low-copy nuclear gene markers have demonstrated prowess in resolving the phylogeny of vascular plants (Zhang et al., 2012; Li et al., 2017). Instead of the summarized hybridizing signal from the gene family tree based on whole-genome or transcriptome analyses, we used low-copy nuclear genes to untangle the reticulate evolutionary history. Many studies have shown that not every gene evolution history can represent the species’ evolutionary history. For instance, some domesticated gene makers only represent domestic history (Huang et al., 2012). We use these universal low-copy nuclear genes to detect more reliable hybridization signals and construct a convenient and efficient method for detecting the authenticity and purity of hybrid plants, which is of great significance for germplasm conservation and crossbreeding.

## Materials and methods

### Data collection: Sequence retrieval of genomes and transcriptomes

We downloaded the *Amborella trichopoda* genome from phytorome12 (http://phytozome.net) and two transcriptomes of Cabombaceae species and 15 other waterlilies from the waterlily genome project to obtain nuclear gene sequences for phylogenetic reconstruction and detection of parent lineages for hybrids (Zhang et al., 2020). The sample represents four genera named *Nymphaea*, *Euryale*, *Victoria*, and *Nuphar* from Nymphaeaceae, two genera called *Brasenia* and *Cabomba* in Cabombaceae, and one outgroup of *Amborella*. Transcriptome assembly was implemented in Trinity v2.8.2 (Grabherr et al., 2011) as described previously (Xiang et al., 2017). Here, we used 15 Nymphaeaceae species, two Cabombaceae species, along with *Amborella trichopoda* as the outgroup for further analyses.

### Selection of putative orthologous genes

Both the ancestors of angiosperm and water lilies have undergone polyploidy events, increasing the challenge of identifying orthologs as phylogenetic markers. Compared to multi-copy nuclear genes, more conserved low-copy nuclear genes were proved effective in resolving angiosperm phylogeny (Zhang et al., 2012). In order to avoid possible biases of specific gene sets and loss of potential effective nuclear gene markers, candidate low-copy marker genes were retrieved from three groups (Figure 2). The first gene set of 931 OGs was acquired based on two orthologous gene databases previously identified from a study of deep angiosperm phylogeny (Zeng et al., 2014). One data set contains 4,180 OGs shared among nine angiosperm species with sequenced genomes (*Arabidopsis thaliana*, *Populus trichocarpa*, *Glycine max*, *Medicago truncatula*, *Vitis vinifera*, *Solanum lycopersicum*, *Oryza sativa*, *Sorghum bicolor*, and *Zea mays*) identified by HaMStR (Deep Metazoan Phylogeny, http://www.deep-phylogeny.org/hamstr/) (Ebersberger et al., 2009). The other data set contains 1,989 low-copy OGs identified from seven angiosperm species with sequenced genomes (*Arabidopsis thaliana*, *Populus trichocarpa*, *Prunus persica*, *Vitis vinifera*, *Mimulus guttatus*, *Oryza sativa*, and *Sorghum bicolor*) identified by OrthoMCL v1.4 (Li et al., 2003) with default parameters. We selected the 931 OGs that were from the intersection of the two databases as phylogenetic markers, representing conserved low-copy OGs across angiosperms. The gene set with 475 OGs selected from 125 Rosaceae species that were identified previously in a deep phylogeny Rosaceae study (Xiang et al., 2017). The third gene set with 852 OGs was obtained from a deep phylogeny Brassicaceae study (Huang et al., 2016a), with high species coverage ratio and gene length. We took an intersection of the gene sets obtained from these three gene pools to remove duplicate genes for subsequent analyses. Finally, 1,167 well-defined putative orthologous genes were used to search for their homologs in 18 flowering plant genomes and transcriptomes using HaMStR (Figures 2, 3). These 1,167 genes were proved as effective gene markers to resolve the angiosperms and Nymphaeales phylogeny with 115 representative species (Zhang et al., 2020). Here, we reconstructed the Nymphaeales phylogeny with only the Nymphaeales representatives and the phylogeny framework was used to infer gene duplication events and hybridization events.

In order to minimize the effects of the hidden paralogues and identify the most probable orthologues, the aforementioned OGs were carefully filtered based on species coverage and gene lengths to identify 1,141 OGs and 942 OGs, respectively. For filtering conditions with a higher species coverage ratio and gene length, two orthologue groups of 882 OGs and 686 OGs were further obtained (see more details in Figure 2).

### Phylogenetic analysis

Multiple sequences of each orthologous group were aligned using MAFFT v7.221 with the “- auto” option (Katoh and Standley, 2013), manually adjusted to remove gaps using MEGA (Kumar et al., 2016). Subsequently, trimAL 1.4 (Capella-Gutiérrez et al., 2009) was used with the “-automated1” option to trim low-quality alignment regions. The phylogenetic relationships were reconstructed by aligning coding sequences to build a maximum likelihood (ML) tree. The coding sequences were aligned using PAL2NAL V14 after being transferred from the protein alignment matrix (Suyama et al., 2006). In this study, ModelFinder (Kalyaanamoorthy et al., 2017) was used to select the best-fit model under the BIC. For phylogenetic reconstruction, the gene sets (1,167 and 834) were analyzed using the coalescence method implemented in ASTRAL v5.5.12 (Mirarab et al., 2014). The 834 OGs selected from 1,167 OGs were more than 840 bp in length and with a species coverage ratio of over 80%. To resolve deep relationships within angiosperm species, it is necessary to eliminate possible noise (e.g., paralogous genes) and avoid system errors caused by the huge super matrix.

### Estimation of divergence time for Nymphaeales phylogeny

Four fossil points were used to calibrate divergence time estimates. The assignments and ages of the fossils include the three Nymphaeales fossils: crown group Nymphaeales with fossils (>121 mya) (Friis et al., 2001), crown group Nymphaeaceae with fossils (>113 mya) (Friis et al., 2009) and crown group Cabombaceae with fossils (>105 mya) (Taylor et al., 2008). In addition, the earliest fossil tricoplate pollen (∼125 mya) associated with eudicots was assigned the minimal original age for crown-group angiosperms (Morris et al., 2018) (Fossils were pinned on the phylogeny in Figure 3).

The four fossil calibrations were implemented as the minimum constraint in our analyses. A Bayesian phylogenomic dating analysis of the 686 selected genes was performed in MCMCtree program from the PAML package (Yang, 2007). The tree topology was confirmed to represent the inferences from our coalescence-based analysis of 942 genes from 18 taxa, using the approximate likelihood calculation to determine branch lengths (Reis and Yang, 2011). Molecular dating was conducted using an auto-correlated model of among-lineage rate variation, the GTR substitution model, and a uniform prior on the relative node times. Posterior distributions of node ages were estimated based on Markov chain Monte Carlo sampling, with samples drawn every 250 steps over 10 million steps, following a burn-in of 500,000 steps. We examined convergence by performing the analysis in duplicate, to ensure sufficient sampling. Date estimates were calibrated using fossil-based age constraints on four tree nodes.

### Processing for multi-labeled gene trees as hybridization signals

HaMStR identified the multiple-hit homologs of 1,167 genes from 18 coding sequence (CDS) of sampled species by not using the “-concat” parameter (Ebersberger et al., 2009). The multi-copy gene family trees were inferred from amino acid converted nucleotide alignment with the GTRGAMMA model by RAxML (Stamatakis, 2006) (Figure 2). A total of 300 multi-copy gene family trees were selected from 1,141 genes with species coverage of more than 50% and retained gene(s) of *Amborella trichopoda* as outgroup for the conversion to multi-labeled gene family.

Overall, the conversion of multi-copy gene family trees into multi-labeled gene family trees follows two principles. The First principle is pruning: Each species should retain one best copy gene (longest) from any paralogs or isoforms, but putative hybrid species/cultivars should be included with multi-copy genes. The second principle is labeling: Label the prefix of the gene ID as A if the hybrid is close to one parental lineage A and Label the prefix of the gene ID as B if the hybrid is close to one parental lineage B. After pruning and labeling, we split the hybrid into two subspecies of representatives, A and B. Finally, we successfully transformed a multi-copy gene tree into a single-copy tree, and each species and labeled subspecies contains one best representative copy. The genes of altered multi-labeled gene family trees were truly orthologous between each other based on the selection from the topology of the gene family tree. The detailed operations of pruning and labeling for multi-labeled gene family trees are described in Figure 2, as illustrated by two examples of multi-copy gene family trees.

### Summarizing hybridization signal for detection of hybridization Event(s)

To identify hybridization signals within the genus *Nymphaea*, 17 sampled water lilies were examined along with basal most species of angiosperm *Amborella trichopoda* as an outgroup. Owing to their unclear speciation and complex breeding history in Nymphaeaceae, *Nymphaea* ‘Choolarp’, *Nymphaea* ‘Paramee’, and *Nymphaea* ‘Thong Garnjana’ were excluded from further analyses. By not using the “-concat” parameter, the multiple-hit homologs of 1,167 genes were identified by HaMStR from 18 CDS of sampled species (Ebersberger et al., 2009). After aligning and trimming the poor regions of the multi-copy matrices, multi-copy gene trees were constructed using RAxML v7.0.4 (Stamatakis, 2006) with the GTRGAMMA model. 17 sampled waterlilies and one outgroup species were divided into nine groups based on the topologies of gene family trees: a (*Nymphaea* ‘Midnight’, *Nymphaea colorata*, *Nymphaea* ‘Woods blue goddess’); b (*Nymphaea* ‘Woods blue goddess’, *Nymphaea caerulea* ‘Savigny’, *Nymphaea* ‘Midnight’); c (*Nymphaea gigantea* ‘Albert de Lestang’, *Nymphaea gigantea* ‘Hybridl’); d (*Nymphaea mexicana*, *Nymphaea tetragona*); e (*Nymphaea potamophila*, *Nymphaea prolifera*, *Nymphaea rubra*); f (*Victoria cruziana*, *Euryale ferox*); g (*Nuphar lutea*, *Nuphar advena*); h (*Cabomba caroliniana*, *Brasenia schreberi*); and i. *Amborella trichopoda*. First, we selected 300 multi-labeled gene family trees with more than 50% taxa coverage and at least one representative for each of the nine aforementioned groups from 1,167 genes. Then, the topologies of the 300 multi-labeled gene family trees were counted and summarized by software phyparts (https://bitbucket.org/blackrim/phyparts/) in Figure 5. Furthermore, based on the condition of monophyly of Nymphaeaceae and Cabombaceae families, 248 and 125 multi-labeled gene family trees were selected for further coalescence and supermatrix ML analyses (detailed pipeline in Figure 2).

## Conclusion

We proposed a novel phylogenomics approach to untangle hybridization or allopolyploidy events by summarizing hybridization signals from multi-labeled gene family trees. The confirmed robust Nymphaeales phylogeny by parsing single-copy and multi-copy gene trees and suggested *Nympheae* possibly is a paraphyletic group and requires a further taxonomic revision for *Victoria* and *Euryale*. We successfully identified two allopolyploidy events with the parental lineages for the hybrids in the family Nymphaeaceae. The well-known cultivars *Nymphaea* ‘Woods blue goddess’ and *Nymphaea* ‘Midnight’ were identified as hybrids and share a common parental progenitor *Nymphaea colarata*. The results coincide with the records of cultivation by horticulturists, further supporting the validity of our proposed phylogenomics approach.

## Data Availability

Publicly available datasets were analyzed in this study. The *Amborella trichopoda* genome were downloaded from phytorome12 (http://phytozome.net). The data of *Nuphar advena* and *Nuphar advena* were downloaded from https://www.ncbi.nlm.nih.gov/ with the accession number of SRX018920 and SRX3469536, respectively. 15 other waterlilies’ data downloaded: https://ngdc.cncb.ac.cn/search/?dbId=&q=PRJCA001283, with the accession number of GWHAAYW00000000 for *Nymphaea colorata*, CRR058886 for *Nymphaea caerulea*, CRR058881 for *Nymphaea* ‘Midnight’, CRR058885 for *Nymphaea* ‘Woods blue goddess’, CRR058888 for *Nymphaea gigantea* ‘Hybridl’, CRR058887 for *Nymphaea gigantea* ‘Albert de Lestang’, CRR058877 for *Nymphaea mexicana*, CRR058878 for *Nymphaea tetragona*, CRR058880 for *Nymphaea potamophila*, CRR058879 for *Nymphaea prolifera*, CRR058876 for *Nymphaea rubra*, CRR058874 for *Euryale ferox*, CRR058875 for *Victoria cruziana*, CRR058889 for *Nuphar lutea*, CRR058873 for *Brasenia schreberi*.
